# 3D Microfluidic Devices in a Single Piece of Paper for the Simultaneous Determination of Nitrite and Thiocyanate

**DOI:** 10.3390/s20154118

**Published:** 2020-07-24

**Authors:** Peng Yu, Muhan Deng, Yi Yang, Beixi Nie, Shaoyu Zhao

**Affiliations:** School of Materials Science and Engineering, Xiangtan University, Xiangtan 411105, China; 201821551419@smail.xtu.edu.cn (M.D.); yangyi@xtu.edu.cn (Y.Y.); 201705721021@smail.xtu.edu.cn (B.N.); 201705721001@smail.xtu.edu.cn (S.Z.)

**Keywords:** three-dimensional microfluidic devices, spraying, colorimetric analysis, nitrite, thiocyanate

## Abstract

The concentrations of nitrite and thiocyanate in saliva can be used as the biomarkers of the progression of periodontitis disease and environmental tobacco smoke exposure, respectively. Therefore, it is particularly necessary to detect these two indicators in saliva. Herein, the three-dimensional single-layered paper-based microfluidic analytical devices (3D sl-μPADs) were, for the first time, fabricated by the spraying technique for the colorimetric detection of nitrite and thiocyanate at the same time. The conditions for 3D sl-μPADs fabrication were optimized in order to well control the penetration depth of the lacquer in a paper substrate. Then, the developed 3D sl-μPADs were utilized to simultaneously detect nitrite and thiocyanate and the limits of detection are 0.0096 and 0.074 mM, respectively. What is more, the μPADs exhibited good specificity, good repeatability, and acceptable recoveries in artificial saliva. Therefore, the developed 3D sl-μPADs show a great potential to determine nitrite and thiocyanate for the assessment of the human health.

## 1. Introduction

Nitrite (NO_2_^−^) widely exists in the environment, food, industry, and physiological systems, which is recognized as a hazardous species [1,2]. Although nitrite intake from food plays an important role in human physiology [3], high amount intake of nitrite is extremely harmful for human health and induces severe health issues [4,5]. Moreover, the nitrate in food can be reduced to nitrite by oral microorganisms [6]. Thiocyanate (SCN^−^) is a substance in nature, which is widely found in the tissues and the secretion of the animals [7]. It is also an important chemical raw material in a lot of industrial sectors, including printing, electroplating, pharmaceutical production, and photofinishing [8]. Furthermore, thiocyanate is a metabolite of hydrogen cyanide found in cigarette smoke [9]. A higher concentration of thiocyanate in physiological fluids can bind to trivalent iron ion in cytochrome oxidase in vivo, inhibiting the enzyme activity and causing hypoxia in human tissue and even obvious damage to renal function. [10]. Both of the two anions (i.e., nitrite and thiocyanate) are normally present in saliva, which are the useful biomarkers for periodontitis disease and tobacco smoke exposure, respectively [11,12]. More importantly, people who often smoke are prone to suffering from oral diseases [13]. Therefore, it is of great importance to simultaneously determine nitrite and thiocyanate in saliva, especially for heavy smokers.

Up to now, many assay techniques have been reported for simultaneously detecting nitrite and thiocyanate, such as capillary electrophoresis [14,15,16], ion chromatography [17,18], isotachophoresis [19], capillary chromatography [20], gas chromatography [21,22], and high-performance liquid chromatography [23]. The above-mentioned techniques are sophisticated with a high sensitivity. However, the limitations for their routine application include poor portability, the high cost, the requirement of large reagent and sample volumes, and cumbersome sample treatment.

Alternatively, point-of-care testing (POCT) has been attracted great interest from researchers, allowing for in-home determinations with the absence of the healthcare professionals [24]. Paper-based microfluidic analytical devices (μPADs) combined with colorimetry make POCT practical in daily life [25]. μPADs possess tremendous advantages including fast detection, low consumption of reagent, good portability, and easy usage [26]. Bhakta et al. quantitatively detected the concentration of nitrite in saliva in a non-invasive way to demonstrate the application value of the μPADs [11]. Pena-Pereira et al. developed the cost-effective μPADs to determine thiocyanate in saliva with low detection limit of 0.06 mM [27]. All the μPADs reported for the detection of nitrite or thiocyanate were all based on two-dimensional (2D) μPADs.

In recent years, three-dimensional (3D) μPADs, especially single-layered configurations, have become a growing research field in μPADs. Compared to the 2D configuration, the fluid in 3D channels can flow in both lateral and vertical directions, making it possible to manipulate complex fluid flow and design complicated chips [28]. Most of the time, 3D μPADs are fabricated by the assemblly of multilayers [29]. The fabrication process is tedious and requires precise stacking and alignment of multiple sheets of paper to construct 3D channels, which hinders the large-scale development of 3D μPADs. To avoid the alignment of paper/tape layers, 3D folding μPADs have been developed and a clamping holder was utilized to firmly fix the multi-layered devices [30]. However, the huge challenges in 3D μPADs manufacturing still exist in mass production in particular. To simplify the fabrication process, 3D single-layered μPADs (3D sl-μPADs) have been reported. Geun Jeong et al. fabricated 3D sl-μPADs by wax printing on double sides and heating the printed patterns by a laminator [31]. Li et al. prepared three-dimensional microfluidic channels by a wax printer in a piece of filter paper [28]. Unquestionably, these studies have made a significant breakthrough in 3D sl-μPADs fabrication. However, they defined the channels with wax, which cannot support the solutions such as surfactants and organic solvents [32]. Therefore, there is still an urgent need to search for a new technology for manufacturing 3D sl-μPADs with high chemical resistance. Furthermore, there have been no reports using the 3D μPADs for the simultaneous detection of nitrite and thiocyanate in saliva so far.

Spraying-based technology has been developed for the fabrication 2D μPADs in recent years. With a comparison to the conventional fabrication techniques, a spraying process displaced the printing process to fabricate 2D μPADs, attracting wide interest of researchers. Nurak et al. constructed 2D μPADs to detect nickel (II) ion by a spray-painting method. The analytical performance of Ni^2+^ assay is very good, and the limit of detection is as low as 8.5 μM [33]. Cardoso et al. fabricated 2D μPADs by the glue-spraying technique. The developed 2D μPADs were highly resistant to chemicals and successfully applied to the colorimetric assays of eight analytes [32]. Our group used the spraying method to deposit patterned adhesive tape in a piece of filter paper to fabricate 2D channels. The developed 2D μPADs provided a simple and cost-effective platform for bioassays [34]. Although these studies have had great breakthroughs in applying the spraying technique to the manufacture of 2D μPADs, there are only a few studies to report the spraying method for fabricating 3D μPADs. Phillips group used the wax-printing technique to make patterned paper and used the glue-spraying method for layers alignment [35]. Their work greatly simplified the assembly steps but still used the wax printer to pattern paper. More importantly, their work was focused on the multi-layered 3D μPADs fabrication. Therefore, there is still a great challenge to apply the spraying method to construct 3D channels in a single piece of filter paper.

In this work, a spraying patterning approach is presented for the fabrication of 3D sl-μPADs relying on double-sided spraying of lacquer. Two important parameters for the fabrication process were optimized: (1) spraying times (the times needed for pressing the spray nozzle), and (2) drying conditions including temperature and time. Under the optimized fabrication conditions, 3D channels were formed in a single piece of paper. The applicability of the fabricated 3D sl-μPADs was demonstrated by simultaneously detecting nitrite and thiocyanate. The results show satisfactory sensitivity, good specificity, good repeatability, and acceptable recoveries in artificial saliva toward nitrite and thiocyanate detection.

## 2. Experimental

### 2.1. Reagents and Instruments

The qualitative filter paper (102#) with medium speed was bought from Hangzhou Fuyang Wood Paper Co., Ltd. (Hangzhou, China) and used for 3D sl-μPADs fabrication. The porosity of the filter paper was 15–20 μm and the ash content of the filter paper was less than 0.15%. The thickness of the filter paper was measured to be 170 μm using a digital vernier caliper. The lacquer (400 mL/can, yellow color) was purchased from Guangdong Haoshun ODIS Technology Co., Ltd. (Zhaoqing, China). The principal component of the lacquer is resin and the lacquer has the advantages of high water resistance and good adhesion. The spraying area is 2.5 m^2^ per can of lacquer. The desired mask was designed using Auto CAD software and cut out from transparent polymethyl methacrylate (PMMA) board with 1 mm in thickness by a local laser cutting shop in China. Black paper binder clips (size 19 mm) were purchased from Deli Co., Ltd. (Ninghai, China) and used to fix the desired mask on the filter paper. Sodium nitrite, potassium thiocyanate, sulphanilamide, N-(1-naphthyl) ethylenediamine dihydrochloride (NED), ferric nitrate nonahydrate, citric acid monohydrate, boric acid, sodium hydroxide, glacial acetic acid, phosphoric acid, sodium carboxymethylcellulose (SCMC) were of analytical grade and purchased from Sinopharm Chemical Reagent Co., Ltd. (Shanghai, China). Poly (acrylic acid) (M.W. ≈3000) was bought from Aladdin-Reagent Company (Shanghai, China). Ultrapure water with electric resistivity of 18.2 MΩ cm was used throughout. The digital photos of the developed assays were taken by a smartphone (MI 8 SE, Beijing Xiaomi Technologies Co., Ltd., Beijing, China) in a foldable photo box. The photo box is equipped with 84 LED lamps and lampshade with the size of 40 × 40 × 40 cm. The distance between the phone and the fabricated 3D sl-μPADs was 8 cm. The digital Red (R), Green (G), and Blue (B) color analysis was done by ImageJ software provided by National Institute of Health (Bethesda, MD, USA).

### 2.2. Solution Preparation

The NO_2_^−^ and SCN^−^ standard stock solutions were made by the dissolvement of NaNO_2_ and KSCN in ultrapure water, respectively. The Griess reagent for NO_2_^−^ detection was made up of 330 mM citric acid, 50 mM sulfanilamide, and 10 mM NED. The Fe (III) reagent solution (600 mM) for SCN^−^ assay was made by the dissolvement of ferric nitrate in Britton–Robinson buffer (pH 7). For the recovery test, the artificial saliva was used as the complex sample, consisting of the following in ultrapure water: NaCl 0.4 g/L; KCl 0.4 g/L; CaCl_2_·2H_2_O 0.8 g/L; NaH_2_PO_4_ 0.69 g/L; Na_2_S·9H_2_O 0.05 g/L; SCMC 0.1 g/L [36,37].

### 2.3. 3D sl-μPADs Fabricated by a Two-Step Spraying Technique

**Figure 1:**
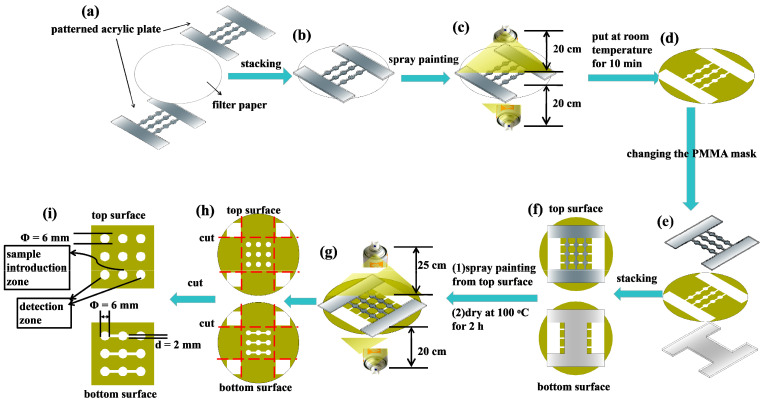
The diagram of the spraying prototyping technique for fabricating the three-dimensional single-layered paper-based microfluidic analytical devices (3D sl-μPADs). (**a**) a sheet of filter paper and two PMMA boards with the same design. (**b**) the paper and two PMMA boards were stacked together. (**c**) the first spraying process. (**d**) the device was dried at room temperature for 10 min. (**e**) the filter paper and two PMMA boards with different designs. (**f**) the paper and two PMMA boards were stacked together. (**g**) the second spraying process. (**h**) the excess materials were trimmed. (**i**) the finished layout of the 3D sl-μPADs.

The spraying prototyping process for fabricating the 3D sl-μPADs was illustrated and shown in Figure 1. A sheet of filter paper was sandwiched between two PMMA boards with the same design (Figure 1a). To fix the relative position between the filter paper and the PMMA boards, the four corners of the PMMA boards were held with four binder clips (Figure 1b). In the first step of the spraying process, the distance between the spray nozzle of the lacquer and the device is set as 20 cm. Then, the spray nozzle of the lacquer was pressed for two times for each side of the filter paper (Figure 1c). After 10 min at room temperature (RT) (Figure 1d), the chip was sandwiched between another two PMMA boards with different designs (Figure 1e). In the second step, the spray nozzle of the lacquer on the top surface is farther from the chip (25 cm) while on the bottom surface the distance between the spray nozzle and the chip is 20 cm (Figure 1g). The spray nozzle of the lacquer was also pressed for two times for each side of the chip. Afterwards, the chip deposited with lacquer was put in a drying chest at 100 °C for 2 h. Finally, excess materials were trimmed (Figure 1h) and the the finished layout of the 3D sl-μPADs were provided (Figure 1i).

### 2.4. Simultaneous Colorimetric Detection of Nitrite and Thiocyanate

The detection principles for nitrite and thiocyanate assays are based on the classical Griess diazo-coupling reaction and the coordination reaction between Fe (III) and thiocyanate, respectively [38,39,40]. Both the two reactions are fast and highly selective. At first, the μPAD was prepared by first adding 1.8 μL of poly (acrylic acid) with a concentration of 0.7 g/L to all the assay reservoirs, waiting for 10 min for drying at RT. After that, 1.8 μL of Griess reagent solution was pipetted to the left detection reservoir while 1.8 μL of the Fe (III) reagent solution was pipetted to the right detection reservoir. Both the reagents were dried at RT for 10 min to form the final sensor. The simultaneous detection of both NO_2_^−^ and SCN^−^ was performed. The detection reservoirs on the left were used for nitrite assay and the detection reservoirs on the right for thiocyanate detection. A total of 18 µL of the standard containing two anions was pipetted into the central reservoir for introducing the sample. After a 15-min waiting period, the image of the device was collected with a phone camera in the professional photography box. The color intensities in the RGB mode were measured by the free software, ImageJ. The whole area of the assay zone was selected, and the mean color value was calculated.

### 2.5. Recovery Test

For the recovery test, the fabrication process of the 3D sl-μPADs was the same as the experimental steps referred to before. The artificial saliva sample was used as the complex sample to detect the recoveries. The addition of SCMC in the artificial saliva was used to mimic human saliva viscosity [41]. The complex sample (18 μL) was spiked with known concentrations of both analytes and was pipetted into the central reservoir afterwards. After waiting for 15 min, the picture of the 3D sl-μPADs was taken by a phone. The color values in the assay zones were measured by the free image processing software.

## 3. Results and Discussion

### 3.1. Conditions Optimization for Spraying Prototyping Process

**Figure 2:**
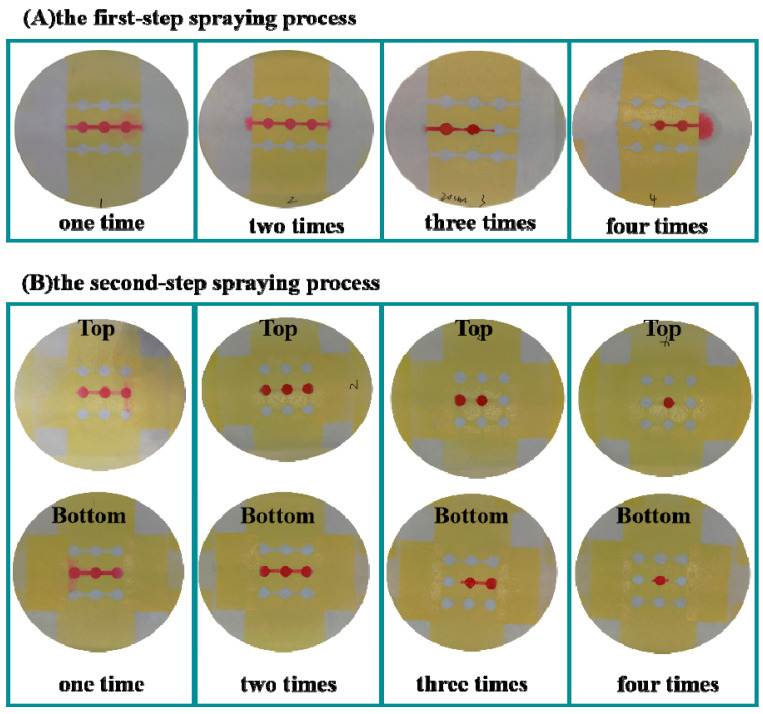
Optimization of the times for pressing the spray nozzle for the spraying process.

**Figure 3:**
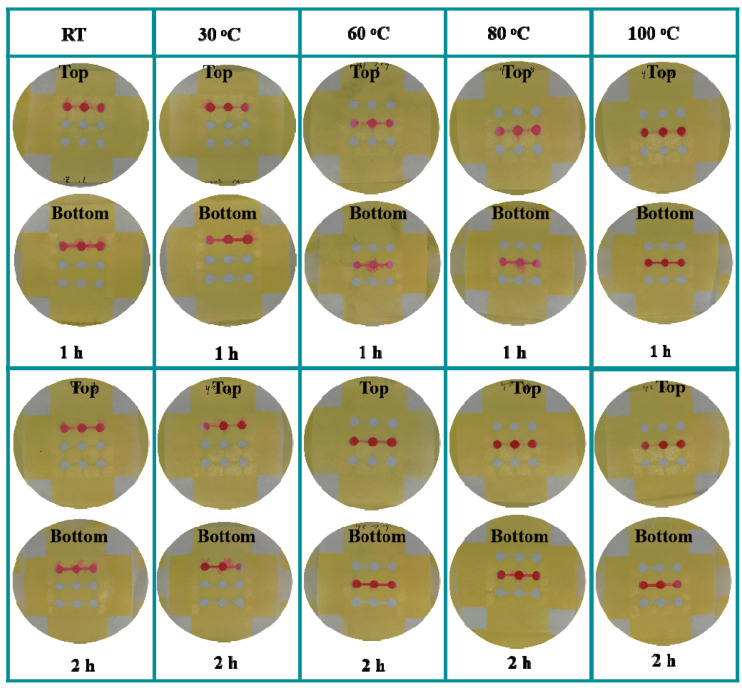
Optimization of the drying conditions for the fabrication of 3D sl-μPADs.

Spraying technique is effective at penetrating the lacquer vertically through the cellulose pore spaces and thereby forming a hydrophobic barrier [33]. Limited and controlled lacquer deposition is extremely important for the fabrication of the 3D sl-μPADs by the spraying prototyping process. To fabricate different patterns on each side of the filter paper, it is necessary to optimize the times for pressing the spray nozzle of the lacquer. For the optimization of pressing times, the developed devices were left at RT for 24 h and the food dye with red color (18 µL) was then added to the central reservoir of the devices. In the first step spraying lacquer, the spray nozzle was pressed different numbers of times. It is noted from Figure 2A that the hydrophobic barrier was not successfully formed after pressing the spray nozzle once. Pressing three and four times results in the excess deposition of lacquer in the filter paper, resulting in narrowing or blocking the hydrophilic channels. Therefore, two times was chosen as the optimized number of times to press the nozzle to ensure the lacquer fully penetrates into the whole depth of the filter paper. In the second step of the spraying process, when the nozzle was pressed two times, the hydrophobic barriers were successfully formed on both the top and bottom sides of the device as shown in Figure 2B. Therefore, two times was chosen for the optimized number of times for the second spraying process. Under the optimized times for pressing the nozzle, the drying temperature and time were also optimized. Several temperatures (RT, 30, 60, 80, and 100 °C) and drying times (1 h, 2 h) were chosen for performing the experiment. The results are shown in Figure 3. It is noted that the lacquer could not be fully dried at low temperature or without enough drying time, resulting in the failure of the desired hydrophobic barrier formation. The optimized drying condition is 100 °C for 2 h, under which the clear hydrophobic boundary was obtained.

### 3.2. Characterization of the 3D sl-μPADs

**Figure 4:**
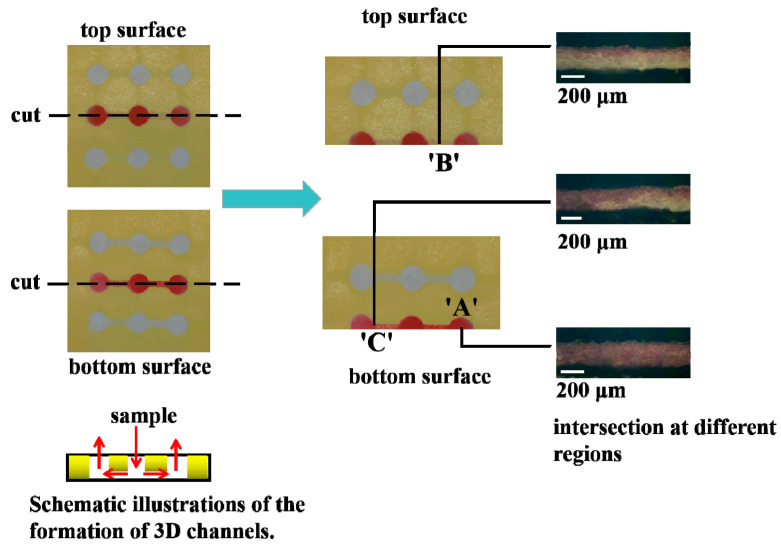
The intersection of the 3D sl-µPADs captured under an optical microscope.

In this work, the 3D sl-μPADs were generated by the spraying prototyping process. To investigate the penetration degree of the lacquer at different regions, the solution containing red food dye (18 µL) was added to the central well of the developed device. Then the solution filtered through the paper, traveled through the filter paper along the channel route, and reached the side wells. Then the fabricated 3D sl-µPADs were cut along the black dotted line after they were fully dried as shown in Figure 4 and the intersection was observed by an optical microscope. It is clearly seen that the paper in spot ‘A’ was un-patterned and the whole depth in spot ‘A’ was hydrophilic. In spot ‘B’, the depth of the yellow lacquer occupied about two-thirds of the whole depth of the device. Therefore, the top side in spot ‘B’ was hydrophobic while the bottom side in spot ‘B’ was hydrophilic. In spot ‘C’, the totally hydrophilic channel and the totally hydrophobic channel were converged together. In addition, the boundary between hydrophobic area and hydrophilic area was very clear. Therefore, it is practicable to control lacquer deposition by optimizing the spraying times and drying conditions.

**Figure 5:**
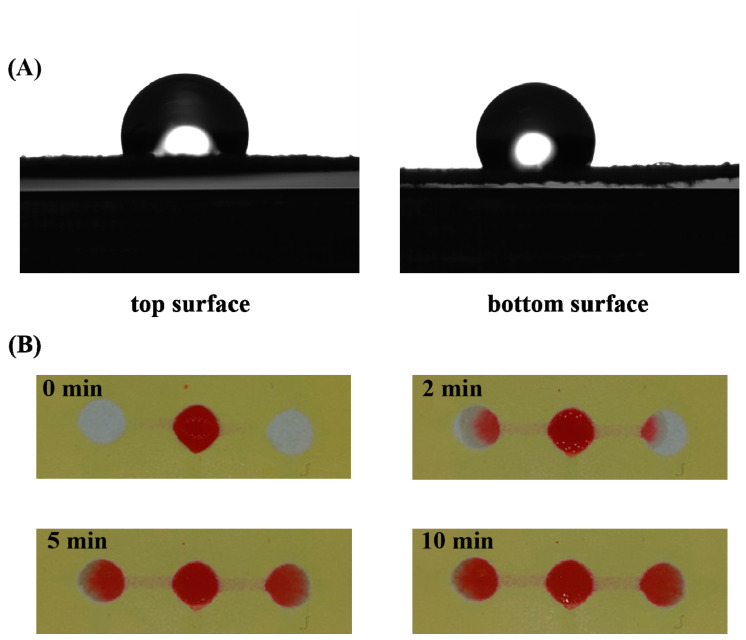
(**A**) The photograph of the water droplet on both sides of the 3D sl-µPADs. (**B**) The pictures are taken at different times after adding food dye (18 µL) to the central reservoir from the top surface of the 3D sl-µPADs.

Furthermore, the wettability behavior of the 3D sl-µPADs was studied. The photographs of water droplet (4 µL) on both sides of the 3D sl-µPADs are shown in Figure 5A. The contact angles with water were 112.77 ± 3.66° for the top surface and 116.21 ± 0.55° for the bottom surface, respectively. The result indicates that the hydrophobic barriers were successfully constructed on both sides of the device by the spraying prototyping process. To demonstrate how the fluid flows in the channel, the food dye with red color (18 µL) was added to the central reservoir on the top surface of the 3D sl-µPADs. The pictures of the 3D sl-µPADs were selected at different time as shown in Figure 5B. The food dye flew through the channel on the bottom surface and then was seen in the left and right detection zones, indicating the successful construction of different patterns on different sides of a single filter paper. The time needed for the fluid flowing from sample introduction zone to both detection zones was 5 min. In addition, the materials cost US $0.0728 per 3D sl-µPAD. It is much lower than the costs of the 3D uPADs fabricated by other groups [42,43,44]. All these results indicate that the spraying method for fabricating 3D sl-μPADs could be an alternative to traditional techniques such as wax printing.

**Figure 6:**
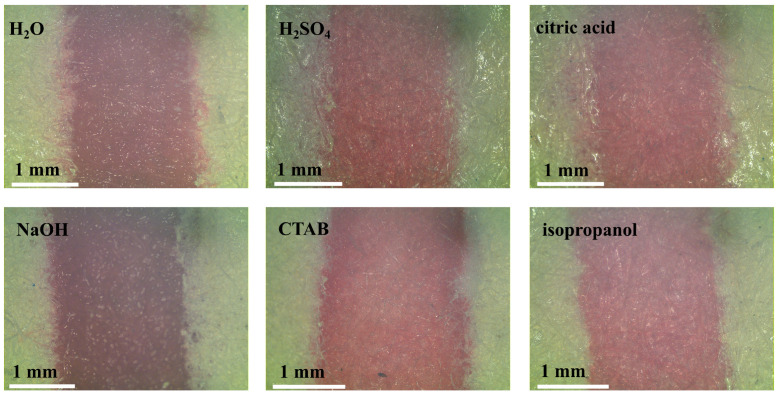
The optical micrographs of the lacquer barrier in the presence of different solutions.

The barrier resistance is one of the important evaluation criteria for μPADs. The chemicals including acid and alkaline solutions, organic solvents, surfactants, etc., were often used in the analytical experiments. Therefore, the resistance of the lacquer barrier towards these chemicals was examined in this work. The solutions used for resistance examination are listed as follows: ultrapure water, acidic solutions (150 mM sulphuric acid, 150 mM citric acid), alkaline solution (150 mM sodium hydroxide), surfactants (5% (*w*/*v*) cetyltrimethylammonium bromide (CTAB)) and organic solvents (50% (*v*/*v*) isopropanol). In order to visually demonstrate the barrier effectiveness, the distance-based μPADs were used with a circular sample introduction zone (8 mm in diameter) and a straight channel (2 mm in width × 25 mm in length) for the resistance experiment. The solution containing a red food dye was pipetted into the sample introduction zone. After drying for 20 min, the optical micrographs of the channels were recorded. The results are shown in Figure 6. There was no solution leakage observed in all the micrographs recorded. The results indicate that the lacquer barrier could support these solutions and exhibited a great compatibility with these solutions. Moreover, the results also indicate that the barrier made by lacquer spraying is better than the wax barrier, because the wax barrier is poorly resistant to the solution such as surfactants and organic solvents [32].

### 3.3. The Feasibility Demonstration

**Figure 7:**
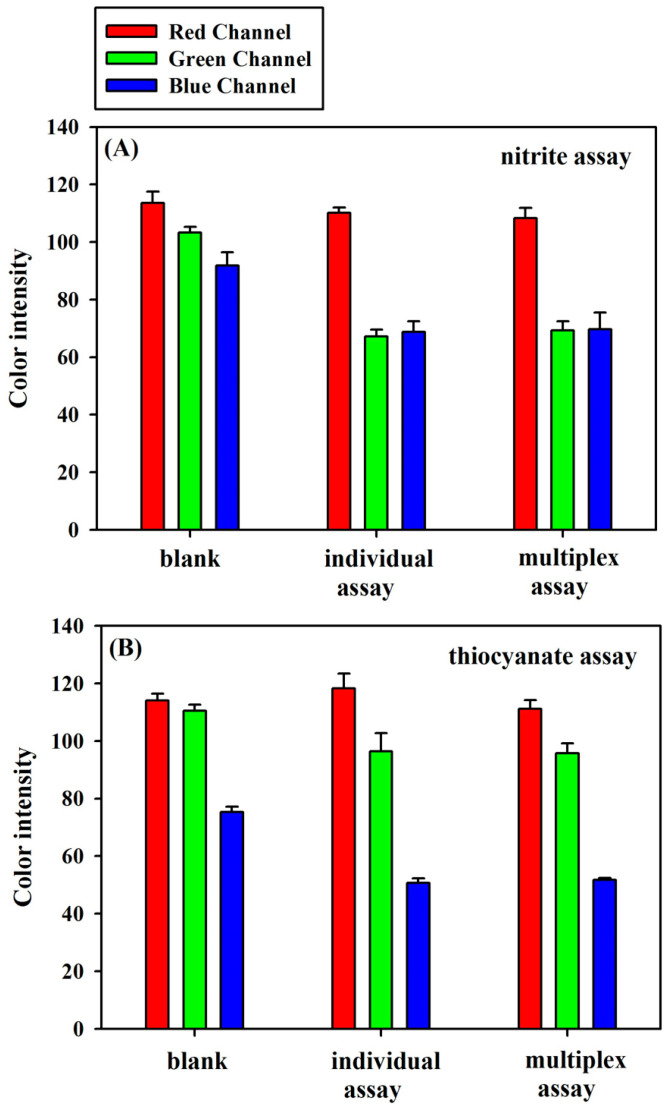
The RGB measurements for the individual assay and the multiplex assay: (**A**) 1 mM nitrite, (**B**) 2 mM thiocyanate.

The color values in the different color channels were measured for demonstrating the feasibility of the 3D sl-µPADs for NO_2_^−^ and SCN^−^ assays. The results for the individual assay and the multiplex assay are shown in Figure 7. For nitrite assay, it is seen that the color values for the R, G, and B channels were 114, 103, and 92 for the blank, 110, 67, and 69 for the individual assay of 1 mM nitrite, and 108, 69, and 70 for 1 mM nitrite in multiplex assay, respectively. The absolute values of the color difference between the blank and the standard for R, G, and B channels were 4, 36, and 23 for the individual assay of nitrite, and 6, 34, and 22 for nitrite in multiplex assay, respectively. Therefore, the color values in the G channel is most sensitive for nitrite assay and chosen as the response signal. For thiocyanate assay, it is seen that the color values for the R, G, and B channels were 114, 110, and 75 for the blank, 118, 96, and 51 for the individual assay of 2 mM thiocyanate, and 111, 96, and 52 for 2 mM thiocyanate in multiplex assay, respectively. The absolute values of the color difference between the blank and the standard for R, G, and B channels were 4, 14, and 24 for the individual assay of thiocyanate, and 3, 14, and 23 for thiocyanate in multiplex assay, respectively. Therefore, the color values in the B channel are most sensitive for thiocyanate assay and chosen as the response signal. Moreover, the color intensities for both nitrite and thiocyanate in individual assay and multiplex assay were almost same. The results indicate that nitrite and thiocyanate could be detected simultaneously and each of them did not interfere with the other’s detection.

### 3.4. Simultaneous Detection of Nitrite and Thiocyanate

**Figure 8:**
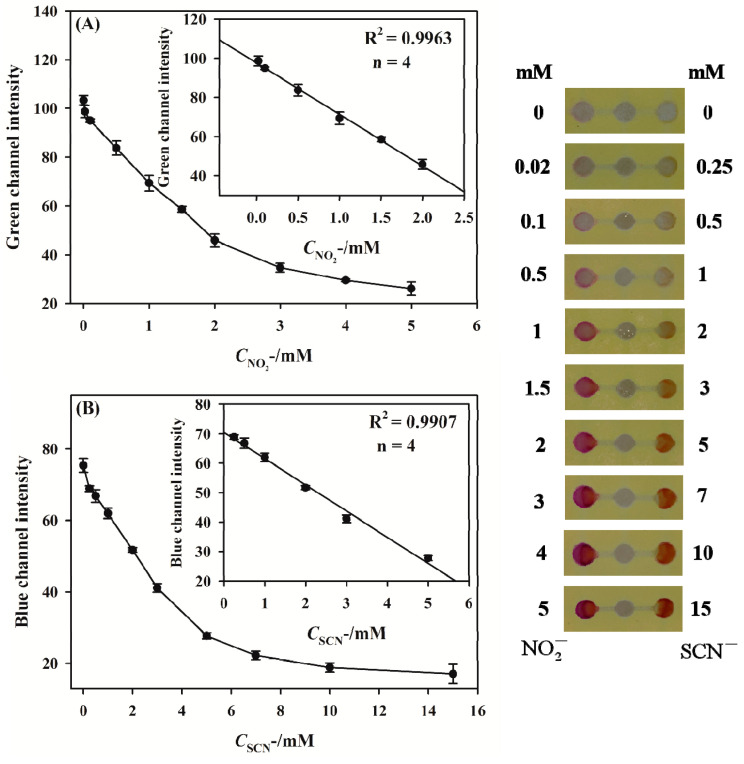
Assay results for nitrite (**A**) and thiocyanate (**B**) based on the developed 3D sl-µPADs.

The simultaneous detection of nitrite and thiocyanate by the 3D sl-µPADs was investigated. The images of the detection zones after the addition of a series of standards are shown in Figure 8. It is noted that the color in the detection zones becomes deeper with increasing nitrite and thiocyanate concentrations from 0 to 5 mM and from 0 to 15 mM, respectively. The changes in color resulted in the notable decreases in the Green channel intensity for nitrite assay (Figure 8A) and the Blue channel intensity for thiocyanate assay (Figure 8B), respectively. Figure 8 shows that the linear ranges are from 0.02 to 2 mM and from 0.25 to 5 mM for nitrite and thiocyanate, respectively. The corresponding linear functions are Green channel intensity = − 26.3098*C*_nitrite_ + 97.6078 (R^2^ = 0.9963) and Blue channel intensity = − 8.9016*C*_thiocyanate_ + 70.4588 (R^2^ = 0.9907) for nitrite and thiocyanate, respectively. The limits of detection (LOD) calculated as the threefold noise level of the blank signal were 0.0096 and 0.074 mM for NO_2_^−^ detection and SCN^−^ detection, respectively. The performance of the developed 3D sl-µPADs was compared with other sensors reported in literature for nitrite and thiocyanate assays as shown in Table 1. Most of the sensors for nitrite or thiocyanate assays mentioned in Table 1 are based on the same reactions and the same analytical method used in this work. Although our 3D sl-µPADs needed the longer time for reaction (15 min) and the bigger volume for sample introduction (18 µL) than some work shown in Table 1, the linear ranges and the LODs obtained in this work were comparable with those given by other sensors. Therefore, the fabricated 3D sl-µPADs provided an analytical platform to quantitatively detect nitrite and thiocyanate at the levels normally found in saliva [45,46,47]. Moreover, a heating step was added to study the possibility of the shorter testing time. The standard containing the two analytes including nitrite (1.5 mM) and thiocyanate (3 mM) was applied for this investigation. After adding the standard in the central well, the device was left in the oven at 50 °C for 3 min. For control, the device without heating treatment was used to detect the same standard with a 15 min assay time. The color intensities were almost the same as those of the control device (data not shown). This indicates that the heating treatment can speed the test duration up to 3 min without losing sensitivity.

### 3.5. Specificity and Precision

**Figure 9:**
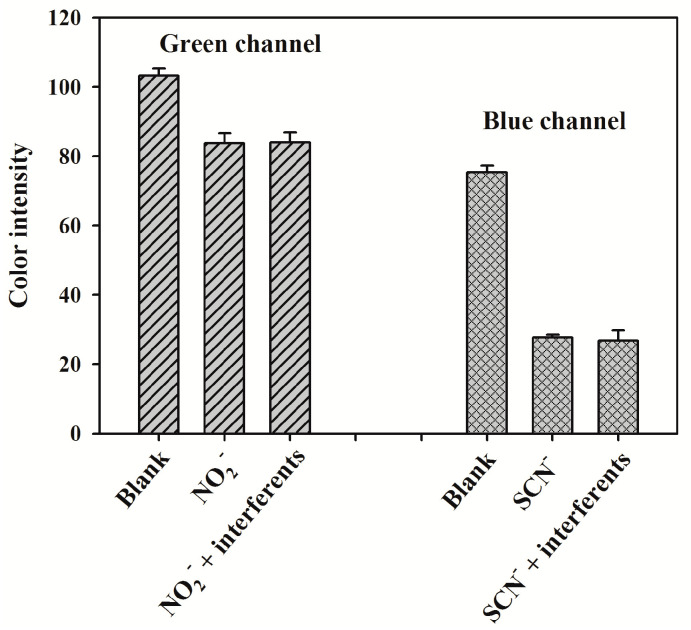
The specificity of the fabricated 3D sl-μPADs. The concentrations of nitrite and thiocyanate are 0.5 and 5 mM, respectively. The concentrations of the interferents are all 50 mM. Error bars represent the standard deviation of four parallel experiments.

The specificity of the developed 3D sl-μPADs was investigated by performing the control experiments using other commonly ions as interferents (NH_4_^+^, CH_3_COO^−^, I^−^, H_2_PO_4_^−^, Cl^−^, SO_4_^2−^, Mg^2+^, Br^−^, NO_3_^−^). Figure 9 shows the color intensity of the detection zones for the specificity experiments. Compared to the blank, the standards containing nitrite and thiocyanate gave the obvious decreases in the Green channel intensity and the Blue channel intensity, respectively. The response signals caused by the mixture of the standards and the interferents are almost the same as those of the standards, indicating the excellent specificity of the developed 3D sl-μPADs toward nitrite and thiocyanate assay. To investigate the precision of this method, the intra-batch and the inter-batch precisions were assessed by the relative standard deviations (RSD) at one concentration level for the multiplex assay: 1 mM nitrite and 2 mM thiocyanate. The intra-batch precision was performed by carrying out eight measurements and the RSD values were 4.5% and 3.9% for nitrite and thiocyanate, respectively. The inter-batch precision was assessed by detecting the same concentration using eight pieces of 3D sl-μPADs from eight different batches. The RSD values for the concentration level were 5.6% and 5.2% for nitrite and thiocyanate, respectively. All these results indicated that the developed 3D sl-μPADs provided a precise assay method for nitrite and thiocyanate detection.

### 3.6. Recovery Test

**Figure 10:**
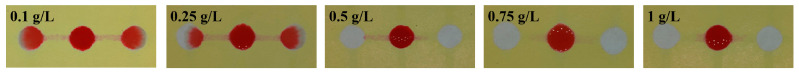
The pictures of the top surface of the 3D sl-μPADs after adding different concentrations of sodium carboxymethylcellulose (SCMC) solutions in the central well.

The practicability of the fabricated 3D sl-μPADs was evaluated by carrying out the recovery test using the artificial saliva as the real sample. The artificial saliva was spiked with the solutions with known concentrations of nitrite and thiocyanate, and was then pipetted into the central reservoir. The photos were acquired after 15 min of sample addition to allow full reaction between the analytes and the reagents. The results of the recovery test in artificial saliva are listed in Table 2. The recoveries for samples with 0.1, 0.5, and 1.5 mM nitrite added and 0.5, 2, and 3 mM thiocyanate added are 110%, 100%, and 95%, and 108%, 92%, and 104%, respectively. Furthermore, the RSD for recovery test was found to be below 6%. Actually, the viscosity of the sample has a great influence on the fluidity of the solution in the channel. SCMC in the artificial saliva was used to mimic the viscosity of the real saliva sample. Different concentrations of SCMC solutions containing a red food dye were used to investigate the solution fluidity in the channel. A total of 18 μL of the SCMC solution was added into the central sample introduction well. After 5 min, the picture of the top surface of the 3D device was taken using a smart phone. As shown in Figure 10, the results demonstrated that the solutions containing 0.1 g/L of SCMC showed the good solution fluidity in the channel. Higher concentrations (larger than 0.25 g/L) of SCMC solutions were too viscous to move in the channel. The viscosity of the artificial saliva used for recovery test is lower than 1.54 mPa·s while the viscosity of the real saliva sample ranges from 1 to 30 mPa·s [41,56]. Therefore, the fluidity of the actual saliva sample in the channel may be a limitation in testing. The pretreatment such as dilution and freezing treatment may be needed before testing since the saliva viscosity was found to decrease significantly after being frozen [57].

## 4. Conclusions

In conclusion, the spraying technique was, for the first time, applied for manufacturing single-layered 3D μPADs. Under the optimized fabrication conditions, 3D channels were successfully constructed in a single piece of filter paper and the hydrophobic boundary was very clear. Furthermore, the developed 3D sl-μPADs were demonstrated for the determination of nitrite and thiocyanate at the same time. The linear ranges are 0.02–2 mM and 0.25–5 mM and the LODs are 0.0096 and 0.074 mM for nitrite and thiocyanate, respectively. Moreover, the 3D sl-μPADs exhibit good specificity, good repeatability, and satisfactory recoveries in the artificial saliva. The analytical performance of the 3D sl-μPADs is sufficient to detect nitrite and thiocyanate at the levels normally found in saliva, indicating their potential applications in POCT. More importantly, through changing the designs of PMMA boards, the 3D sl-μPADs could accommodate more complex channels and enable high-throughput detection.

## Figures and Tables

**Table 1:** A contrast of the assay performance between our 3D sl-µPADs and other sensors reported by other groups in the determination of nitrite and thiocyanate.

**Nitrite Assay**						
**Sensing Reagents**	**Method**	**Response Time (min)**	**Volume (μL)**	**Linear Range (mM)**	**LOD (mM)**	**Refs.**
Griess reagent	Colorimetric	2	15		0.16	[48]
Griess reagent	Colorimetric				0.087	[49]
Griess reagent	Colorimetric	2	1.40	0.156–1.25		[50]
Griess reagent	Colorimetric	10	5	0.0156–1	0.0148	[51]
Griess reagent	Colorimetric	10	12	0.02–0.9	0.015	[36]
Griess reagent	Colorimetric	15	18	0.02–2	0.0096	This work
**Thiocyanate Assay**						
**Sensing Reagents**	**Method**	**Response Time (min)**	**Volume (μL)**	**Linear Range (mM)**	**LOD (mM)**	**Refs.**
Fe^3+^	Colorimetric	4	15	1–8	1.19	[52]
Fe^3+^	Colorimetric	10	2	0.025–20	0.06	[27]
Fe^3+^	Colorimetric			0.10–0.34	0.10	[53]
Fe^3+^	Colorimetric		100	2.55–25.48	0.20	[54]
Astra phloxine	Spectrophotometic		50	0.05–0.50	0.02	[55]
Fe^3+^	Colorimetric	15	18	0.25–5	0.074	This work

**Table 2:** Recovery test of nitrite and thiocyanate in artificial saliva.

**Nitrite**	**Sample No.**	**Added (mM)**	**Found (mM)**	**RSD (%)**	**Recovery (%)**
	1	0.1	0.11	5.63	110
	2	0.5	0.50	1.98	100
	3	1.5	1.43	1.88	95
**Thiocyanate**	**Sample No.**	**Added (mM)**	**Found (mM)**	**RSD (%)**	**Recovery (%)**
	1	0.5	0.54	2.71	108
	2	2	1.83	2.30	92
	3	3	3.13	2.84	104
